# Associations between Physical Activity and Liver Cancer Risks and Mortality: A Systematic Review and Meta-Analysis

**DOI:** 10.3390/ijerph17238943

**Published:** 2020-12-01

**Authors:** Junga Lee

**Affiliations:** Sports Medicine and Science, Kyung Hee University, Global Campus 17104, Korea; jalee@khu.ac.kr

**Keywords:** lung cancer, physical activity, mortality, meta-analysis

## Abstract

(1) Background: The purpose of this meta-analysis was to investigate associations between physical activity (PA) and risks and mortality of liver cancer (LC) to suggest a minimum physical activity threshold to reduce LC risks and morality. (2) Methods: A database search was performed to identify relevant studies on the associations between PA and risks and mortality of LC before August 2020. The PA amounts were divided into three groups (high: ≥3 h/week, moderate: 2–3 h/week, and low: <2 h/week). The pooled relative risks of LC were calculated. (3) Results: A total of 10 prospective cohort studies were included. LC risks and mortality were 26% and 25% lower with high amounts of PA and 23% and 19% lower in moderate amounts of PA, respectively, compared to low amounts of PA. At the vigorous intensity PA level, high and moderate amounts of PA reduced the LC risk by 54% and 45%, respectively. (4) Conclusions: PA helps to reduce LC risks and mortality in a dose-dependent manner. At a minimum, two hours/week PA are mandatory to reduce LC mortality.

## 1. Introduction

Liver cancer is the fourth leading cause of death worldwide and accounts for about 782,000 deaths in the global population [1]. The mortality of liver cancer has increased by about 65% in the last two decades, during which obesity, diabetes, and physical inactivity have increased [2]. The prognosis for liver cancer survival is worse than for other cancers; finding effective behaviors to prevent liver cancer will be important for patients [3,4]. Increasing physical activity may play a pivotal role in reducing the associated risks and mortality of liver cancer because of the beneficial effects of physical activity, which can reduce adiposity in the body, insulin levels, insulin resistance, and inflammation factors, which can improve lipid profiles [5,6,7].

Although several cancer studies including studies on breast cancer, colorectal cancer, lung cancer, prostate cancer, and ovarian cancer have reported inverse associations between physical activity and risks and mortality for all cancers, only a few studies have investigated these associations in liver cancer [8,9,10,11]. Additionally, the association between physical activity and cancer mortality had a dose-response with physical activity [12,13] and those associations were dependent on physical activity intensity [14]. Regarding liver cancer, a previous meta-analysis reported an inverse association between daily total physical activity and risk of liver cancer in two studies, and did not find a significant association between leisure physical activity time and risks of liver cancer in five studies, which showed the associations between physical activity type, including daily total physical activity, leisure physical activity, and liver cancer risk [15]. Another previous meta-analysis demonstrated an inverse association between physical activity and risks of liver cancer in eight studies, which reported the associations between total physical activity and liver cancer risk regardless of physical activity amount, intensity, and type. Associations between liver cancer mortality and total physical activity were inversely associated in three studies [16]. The amount, intensity, and type of physical activity influenced liver cancer risk and mortality. To date, no meta-analysis has assessed the association between liver cancer risks and mortality and physical activity based on the dose-response of physical activity. Because the amount of physical activity can impact chronic disease factors, the association between liver cancer risks and mortality and the intensity of physical activity should be investigated. Therefore, the purpose of this meta-analysis was to investigate the associations between physical activity and liver cancer risks and mortality, depending on the amount of time and intensity of physical activity. In addition, determining benefits of physical activity, including the minimum amount of time and intensity of physical activity, can provide guidance for reducing liver cancer risks and mortality.

## 2. Materials and Methods

### 2.1. Eligible Studies

The Preferred Reporting Items for Systematic Reviews and Meta-analyses (PRISRM) system was used for this meta-analysis [17]. Eligible studies published in English from January 2007 to August 2020 were found using the PubMed and EMBASE databases. Several key terms used in the search to select relevant studies were: “liver cancer,” “physical activity,” “exercise,” “risk of liver cancer,” “liver cancer incidence,” “liver cancer mortality,” “liver cancer death,” and “liver cancer survival”. Possible combinations of these key terms were used to find all relevant studies. Cited references were reviewed to estimate any additional relevant studies. A researcher and a reviewer searched relevant studies independently based on inclusion and exclusion criteria. Any disagreements about selected studies between the researcher and reviewer were discussed to come to an agreement. All relevant studies followed our inclusion and exclusion criteria. Reporting the relative ratio (RR) or hazard ratio (HR) of associations between physical activity and risks and mortality of liver cancer, presenting physical activity assessments, criteria for cause of death, and being a prospective cohort study were required for inclusion. Reviewed studies, pilot studies, protocol studies, and studies without a RR or HR were excluded.

### 2.2. Data Extraction

The basic characteristics of selected studies, including the name of the first author, year of publication, the name of cohort studies, study design, country where the study was conducted, sample size, follow-up period, criteria for the risks and mortality of liver cancer, assessments of physical activity, RR or HR, 95% CIs, and adjusted values for each study were extracted. All information from the studies is presented in Table 1, based on the guidelines for the Meta-analysis of Observational Studies in Epidemiology.

### 2.3. Statistical Analysis

A summary of the RR with 95% confidence intervals (CI) across the selected studies was computed to find the associations between physical activity and risks and mortality of liver cancer. The amounts of physical activity were divided into three groups (high: ≥3 h/week, moderate: 2–3 h/week, and low: <2 h/week). The averages of the three groups that were directly extracted from the selected studies were used as cut-off points. If the summary of the RR was a heterogeneous model (*p* > 0.1), a random-effects model was used. If the summary of the RR was homogenous (*p* < 0.1), a fixed-effects model was used. Heterogeneity across sampled studies was determined by using the Q statistic. Inconsistency across the sampled studies was detected by using the I^2^ statistic. Publication bias was determined by using the Begg and Egger tests and a visual inspection of the funnel plot. All statistical analyses were conducted with a comprehensive meta-analysis version 1.25 software program (Biostatic, Inc., Englewood, NJ, USA).

## 3. Results

**Figure 1:**
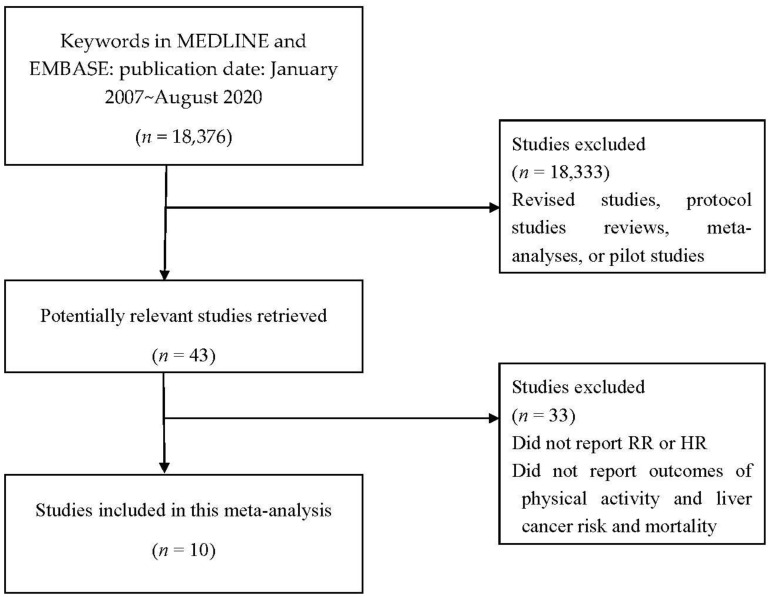
Selection process for the systematic review and meta-analysis.

The selection processes for this meta-analysis are detailed in Figure 1. A total of 18,376 studies were found in an initial search. From this total, 18,333 studies were excluded based on title and abstract screening. The full texts of the remaining 43 studies were reviewed, and 33 studies that did not report an RR or HR for associations between physical activity and risks and mortality of liver cancer were excluded. Finally, 10 studies that met the criteria for this meta-analysis were selected [18,19,20,21,22,23,24,25,26,27].

Ten prospective cohort studies presented in Table 1. Four studies were conducted in the United States of America. One multi-nation study included subjects in Denmark, France, Germany, Greece, Italy, the Netherlands, Norway, Spain, Sweden, and the United Kingdom. Three studies were performed in Japan, one study was conducted in Taiwan, and one study was performed in the Republic of Korea. Physical activity was measured by physical activity questionnaires. In total, 8,283,426 subjects are included in this meta-analysis. An average follow-up was 11.35 years and ranged from 6 to 19.4 years. The amount of physical activity was pooled from each study, which indicated whether the amount was high, moderate, or low. A high amount of physical activity was defined as an average of ≥3 h per week, a moderate amount of physical activity as 2 to 3 h per week, and a low amount of physical activity as <2 h per week.

### 3.1. Associations between Physical Activity and Liver Cancer Risks

**Figure 2:**
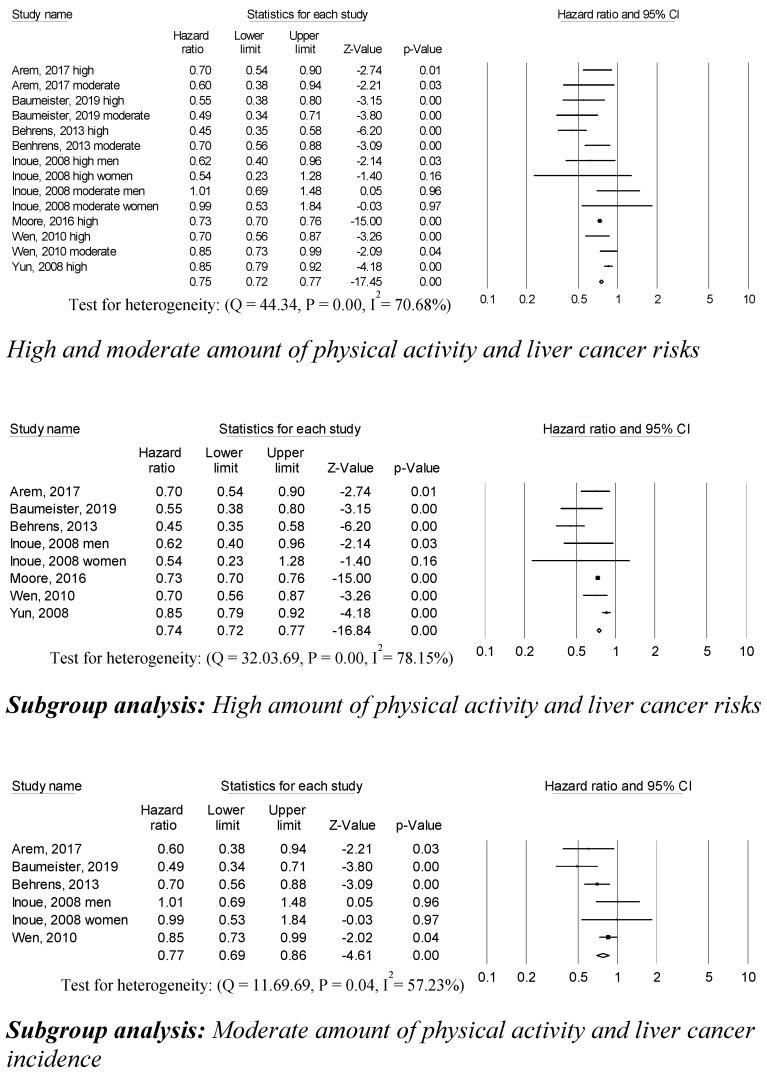
Physical activity and liver cancer risks.

High and moderate amounts of physical activity were associated with a 75% decreased risk of liver cancer (0.75 (95% CI, 0.72–0.77; *p* = 0.001)) compared to low amounts of physical activity in fourteen studies (Figure 2). Subgroup analyses categorized by high and moderate amounts of physical activity were conducted. Eight trials were included to analyze the association between a high amount of physical activity and liver cancer risks. Individuals who participated in high amounts of physical activity had lower liver cancer risks compared to those who participated in low amounts of physical activity (0.74 (95% CI, 0.72–0.77; *p* = 0.001)). The association between a moderate amount of physical activity and liver cancer risk was computed based on six studies. A moderate amount of physical activity was associated with lower cancer risk compared to those who participated in low amounts of physical activity (0.77 (95% CI, 0.69–0.86; *p* = 0.001)). Statistically significant heterogeneity across sampled studies was found, based on the association between high and moderate amounts of physical activity and liver cancer risks. No publication bias or any apparent influence of unpublished data were detected by using the trim and fill method.

### 3.2. Associations between Vigorous Intensity and Amount of Physical Activity and Liver Cancer Risk

**Figure 3:**
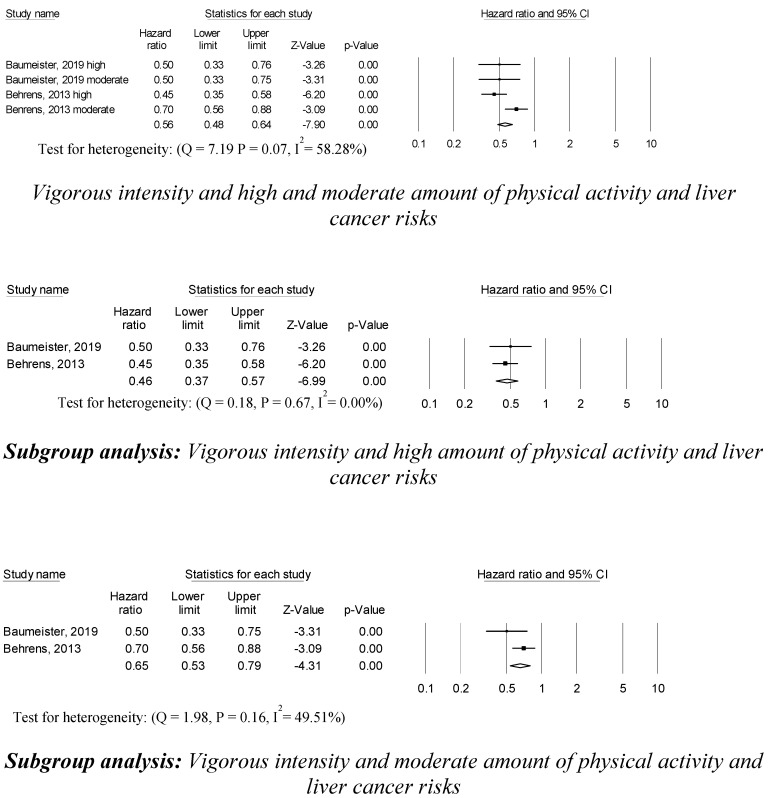
Vigorous intensity and moderate amount of physical activity, and liver cancer risks

Four studies reported that vigorous intensity and high and moderate amounts of physical activity were associated with a 56% decreased risk of liver cancer (0.56 (95% CI, 0.48–0.64; *p* = 0.001)) compared to vigorous intensity and low amounts of physical activity (Figure 3). Subgroup analyses were conducted by the amount of vigorous intensity physical activity regimens. In two studies, vigorous intensity and high amounts of physical activity were associated with a 44% lower risk of liver cancer (0.46 (95% CI, 0.37–0.57; *p* = 0.001)) than vigorous intensity and low amounts of physical activity. In addition, in two studies, vigorous intensity and moderate amounts of physical activity were associated with a 35% lower risk of liver cancer (0.65 (95% CI, 0.53–0.79; *p* = 0.001)) than vigorous intensity and low amounts of physical activity. No statistically significant heterogeneity across the sampled studies was detected in the vigorous intensity and both the high and moderate amounts of physical activity and liver cancer risk studies. The trim and fill method revealed no publication bias or any apparent influence of unpublished data.

### 3.3. Associations between Physical Activity and Liver Cancer Mortality

**Figure 4:**
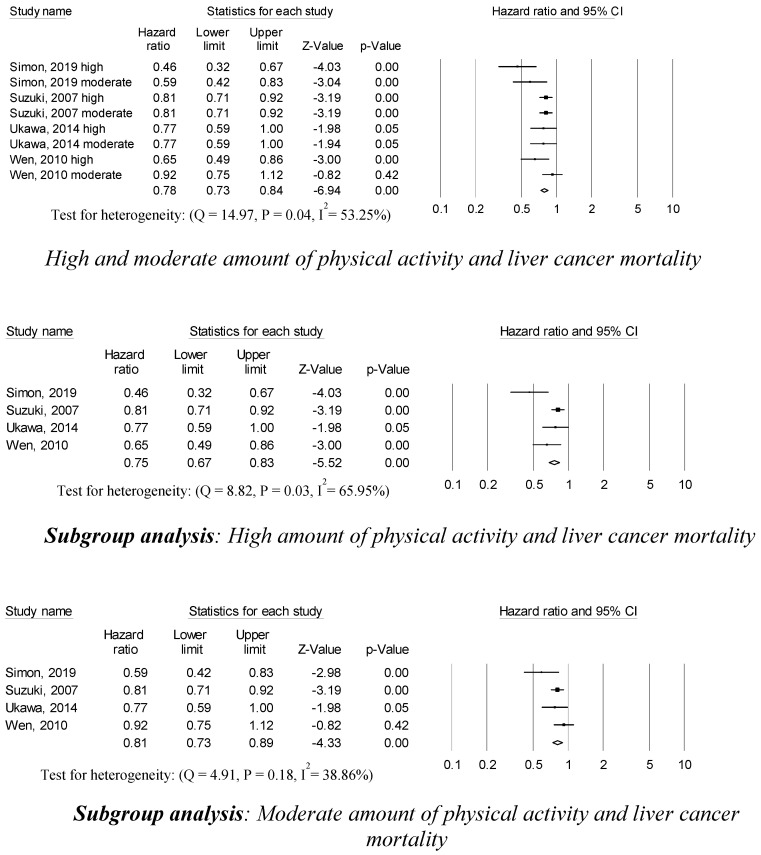
Physical activity, and liver cancer mortality.

Eight studies reported that participating in high and moderate physical activity was associated with a 78% decreased risk of liver cancer mortality (0.78 (95% CI, 0.73–0.84; *p* = 0.001)) compared to low amounts of physical activity (Figure 4). Participants with high amounts of physical activity had a 25% reduced liver cancer mortality compared to participants with low amounts of physical activity (0.75 (95% CI, 0.67–0.83; *p* = 0.001)) in four studies. Moderate physical activity was associated with a 19% decrease in liver cancer mortality (0.81 (95% CI, 0.73–0.89; *p* = 0.001)) in four studies. Statistically significant heterogeneity across sampled studies was found in the association between high amounts of physical activity and liver cancer mortality, but no heterogeneity across sampled studies was found in the association between moderate amounts of physical activity and liver cancer mortality. There was no publication bias or any apparent influence of unpublished data based on the trim and fill method.

## 4. Discussion

Risks and mortality of liver cancer were associated with the amount and intensity of physical activity. High (≥3 h per week) and moderate amounts (2 to 3 h per week) of physical activity were associated with lower risks and mortality of liver cancer compared to low amounts (<2 h per week) of physical activity. Considering the exercise intensity, the selected studies in this meta-analysis only reported associations between vigorous intensity and high, moderate, and low amounts of physical activity and risks of liver cancer. Physical inactivity was associated with increased liver cancer mortality. More than 2 h per week of physical activity, which was more than the low amount of physical activity (<2 h per week) used as a reference, may help reduce the risk and mortality associated with liver cancer.

Participating in physical activity had an inverse association with liver cancer risks. Depending on the amount of physical activity, high amounts of physical activity were associated with a 26% decreased risk of liver cancer. In addition, a moderate amount of physical activity was associated with a 23% decreased risk of liver cancer. These findings are in agreement with previous meta-analyses [12,16,28]. The type of physical activity such as leisure-time physical activity and walking was not specified in this meta-analysis because of the limited number of selected studies; therefore, evaluation of specific types of activity are needed in future studies. The associations between high and moderate amounts of physical activity and liver cancer risks had statistically significant heterogeneity across sampled studies in this meta-analysis; therefore, these values could be associated with types and intensities of physical activity. Analysis of physical activity intensity in this meta-analysis did not allow for comparison with other intensities, specifically moderate intensity and low intensity physical activity because studies only reported vigorous intensity with high, moderate, and low amounts of physical activity. More than 2 h per week of physical activity was congruent with a recent study that recommended at least 2.1 h per week of leisure-time physical activity as well as suggested that there are inverse relationships between physical activity intensity and liver cancer risk [13]. A sensitivity analysis of vigorous intensity indicated that individuals who engaged in high amounts of physical activity had a 54% decreased risk of liver cancer and subjects that did moderate amounts of physical activity had a 35% decreased risk of liver cancer compared to low amounts of physical activity. Vigorous intensity and high amounts of physical activity were associated with the lowest risk of liver cancer in this meta-analysis. There was no statistically significant heterogeneity across sampled studies in vigorous intensity and high and moderate amounts of physical activity, although the analyses were only conducted for vigorous intensity.

Liver cancer mortality was decreased by 22% in individuals who performed high and moderate amounts of physical activity, by 25% in individuals who performed high amounts of physical activity, and by 19% in participants who performed moderate amounts of physical activity. Reduced liver cancer mortality in this current meta-analysis was similar to a previous meta-analysis that conducted a sensitivity analysis of three studies that reported a 20% decrease in liver cancer mortality in individuals performing high amounts of physical activity compared to low amounts of physical activity [16]. A previous meta-analysis reported that reduced all-cause mortality was associated with participation in high amounts of physical activity [12]. Additionally, a previous study reported reduced liver cancer mortality in those who performed at least 90 min per week of physical activity [26], and pre-diagnosis moderate to vigorous intensity leisure-time physical activity was associated with a 29% reduced risk of liver cancer mortality [29]. The current findings for the associations between physical activity and liver cancer mortality do not consider intensity and type of physical activity; therefore, those results may be associated with statistically significant heterogeneity across sampled studies. Although liver cancer mortality was decreased by participating in high and moderate amounts of physical activity, additional studies that consider the type and intensity of physical activity are needed to confirm these findings.

There are several possible mechanisms for the beneficial effects of physical activity on liver cancer risks and mortality. First, physical activity leads to decreased hyperglycemia, which may be associated with reduced growth-promoting and mitogenic activity of cancer cells and carcinogenesis by increasing insulin-like growth factor [30,31,32]. In addition, decreased insulin resistance can lead to lower levels of proinflammatory cytokines, including tumor necrosis factor-alpha, interleukin-6, leptin, and chronic hepatic inflammatory markers, which have been associated with liver diseases including liver cancer [33,34,35,36]. Third, the beneficial effects of physical activity include decreased adiposity, including visceral adipose tissue volume, which has been associated with the progression of fibrosis, inflammation, and liver fat [37]. Finally, increased physical activity may improve circulation, immune function, energy balance, and insulin sensitivity, which influence and can reduce the associated risks of liver cancer [5,23,25].

This meta-analysis was strengthened by the dose-dependent analyses of the amount of time of physical activities that were included in prospective cohort studies, and by considering the intensity of physical activity. However, there are several limitations to this meta-analysis. First, although the populations of the selected studies were large, the total number of selected studies for this meta-analysis was only 10; therefore, generalizations are limited. Second, the findings of this meta-analysis do not support the causality of the associations between physical activity and liver cancer risks and associated mortality; therefore, the findings should be considered carefully. Third, physical activity was measured using self-reported questionnaires. More objective measurements including accelerometers should be used in future studies. In addition, physical activities including walking, leisure-time activities, and other activities should be specified to assess the impact of specific types of activity. Fourth, the RR or HR for each study in selected studies was adjusted for different factors such as age and sex, which could influence the associations in this meta-analysis. Finally, time points for measuring physical activity were evaluated prior to a diagnosis of liver cancer; therefore, there is a possibility that the physical activity patterns could change after the liver cancer diagnosis.

## 5. Conclusions

In this meta-analysis, participating in physical activity had beneficial effects including reduced risk and death in liver cancer patients. Considering physical activity intensity, type, and amount, additional studies need to confirm these findings. Recommendations for liver cancer patients suggest that regular physical activity for more than 2 h per week may be a minimum physical activity level that can help reduce liver cancer risks and mortality. Even for vigorous physical activity intensity, high amounts of physical activity, which showed the lowest mortality values for liver cancer compared with moderate and low amounts of physical activity, may be more favorable than moderate and low amounts of physical activity for preventing liver cancer risks. Although more studies need to be conducted, guidelines for participating in physical activity for more than 2 h per week at vigorous intensity may help improve patient outcomes.

## Figures and Tables

**Table 1:** Characteristics of selected studies: physical activity and liver cancer risks and mortality.

First Author (Year), Name of Sudy, Country	Sample Size Study, Recruitment Period, and Age	Follow-Up Period (Year)	Criteria of the Cause of Death	Exercise Assessment	Relative Ratio (95% CI) Exercise: Metabolic Equivalent of Task (MET)/Hour/Week		Adjusted for
Arem (2017), the NIH-AARP Diet and Health study cohort, U.S.A.	566,398 (1995–1997), 50–71 years old	Median: 13.1 years	Hepatocellular carcinoma (HCC) with morphology code, and cancer registry case ascertainment	Physical activity questionnaire	**Liver cancer incidence**		Sex, and age
					Low	1	
					High	0.70 (0.54, 0.90)	
					Met recommendations	0.60 (0.41, 1.01)	
Baumeister (2019), Multinational cohort study, 10 countries (Denmark, France, Germany, Greece, Italy, the Netherlands, Norway, Spain, Sweden and the United Kingdom)	467,336 (1992–2000), mean age 51.3 (9.9) years	Mean: 14.9 years	International Classification of Diseases-Oncology-2, HCC	Physical activity questionnaire	**Liver cancer incidence** **Total physical activity**		Education, smoking baseline alcohol consumption, lifetime alcohol, and coffee intake
					Inactive	1	
					Moderately Inactive	0.65 (0.48, 0.89)	
					Moderately active	0.49 (0.34, 0.71)	
					Active	0.55 (0.38, 0.80)	
					**Vigorous physical activity**		
					None	1	
					≤2 h/week	0.50 (0.33, 0.75)	
					>2 h/week	0.50 (0.33, 0.76)	
Behrens (2013), the NIH-AARPDiet and Health Study, U.S.A.	4,604,015 (1995–2006), mean age 62(5.4)	Median: 9.1 years	North American Association of Central Cancer Registries, and International Classification of Diseases for Oncology (ICD-O-3)	Physical activity questionnaire	**Liver cancer incidence** **Vigorous physical activity**		Age and sex
					0	1	
					<1	0.73 (0.56, 0.95)	
					1–2	0.70 (0.56, 0.88)	
					2–3	0.54 (0.43, 0.68)	
					5+	0.45 (0.35, 0.58)	
Inoue (2008), the Japan Public Health Center-based Prospective Study, Japan	79,771 (1995–1999), aged 45–74 years	7.5 years	International Classification of Diseases	Physical activity questionnaire	**Liver cancer incidence** **Total physical activity** **Man**		Age, total energy intake, history of diabetes, smoking status, never smoker, past smoker, alcohol intake status, body mass index (BMI), leisure-time sports or physical exercise
					Lowest	1	
					Second	0.69 (0.45, 1.06)	
					Third	1.01 (0.69, 1.49)	
					Highest	0.62 (0.40, 0.96)	
					**Women**		
					Lowest	1	
					Second	0.96 (0.52, 1.78)	
					Third	0.99 (0.53, 1.84)	
					Highest	0.54 (0.23, 1.29)	
Moore (2016), the Physical Activity Collaboration of the National Cancer Institute’s Cohort Consortium, U.S.A.	1,440,000 (1987–2004), the median age 59 years	A median 11 years	The international Classification of Diseases for Oncology	Physical activity survey	**Liver cancer incidence** **Leisure-time physical activity**		BMI
					Lower	1	
					Higher	0.73 (0.70, 0.76)	
Simon (2019), the Nurses’ Health Study and the Health Professionals Follow-up Study, U.S.A.	125,264 (1986–1989), aged 30–55 years	12 years	National Death Index, ICD-8, HCC	Physical activity questionnaire	**Liver cancer mortality**		Age
					Lowest	1	
					Second	0.70 (0.51, 0.96)	
					Third	0.59 (0.42, 0.84)	
					Fourth	0.52 (0.36, 0.74)	
					Highest	0.46 (0.31, 0.78)	
Suzuki (2007), the Japan Collaborative Cohort Study, Japan	69,752 (2005–2011), aged 40–79 years	12 years	ICD-10	Physical activity questionnaire	**Liver cancer mortality**		Age, sex, area
					<1 h/week	1	
					>3 h/week	0.81 (0.71, 0.92)	
					**Walking time (per day): men**		
					>1 h/day	1	
					0.5–1 h/day	0.81 (0.71, 0.92)	
					<0.5 h/day	1.43 (1.10, 1.86)	
					**Walking time (per day): women**		
					>1 h/day	1	
					0.5–1 h/day	1.03 (0.67, 1.65)	
					<0.5 h/day	1.84 (1.27, 2.66)	
Ukawa (2014), the Japan Collaborative Cohort Study, Japan	69,752 (1998–1990), aged 40–79 years	19.4 years	International Classification of Diseases, Ninth Revision, Clinical Modification	Physical activity questionnaire	**Liver cancer mortality** **Walking time** **Man and Women**		Age, sex, study area, smoking status, alcohol consumption, daily consumption of coffee, BMI, education level, marital status, a history of diabetes mellitus, gall bladder diseases, blood transfusion
					≤0.5 h/day	1	
					>0.5 h/day	0.77 (0.59, 0.99)	
					**Man**		
					≤0.5 h/day	1	
					>0.5 h/day	0.81 (0.58, 1.14)	
					**Women**		
					≤0.5 h/day	1	
					>0.5 h/day	0.70 (0.47, 1.07)	
Wen (2010), Health Management Institution of prospective cohort study, Taiwan	416,175 (1976–2007), aged 20–79 years	8.5 years	National Death file and the National Cancer Registry file	Physical activity questionnaire	**Liver cancer mortality**		Age, sex, education, activity at work, smoking, drinking, fasting blood glucose, systolic blood pressure, BMI, diabetes history, hypertension, history
					Inactive	1	
					Low	0.97 (0.80, 1.18)	
					Medium	0.92 (0.75, 1.12)	
					High	0.80 (0.60, 1.07)	
					Very high	0.65 (0.49, 0.86)	
					Total	0.85 (0.72, 0.99)	
					**Liver cancer incidence**		
					Inactive	1	
					Low	0.95 (0.82, 1.10)	
					Medium	0.85 (0.73, 1.00)	
					High	0.87 (0.70, 1.08)	
					Very high	0.70 (0.56, 0.86)	
					Total	0.81 (0.71, 0.92)	
Yun (2008), National Health Insurance Corporation Study, Republic of Korea	444,963 (1996–2002) mean age 49 (6.5) years old	6 years	Korean Central Cancer Registry	Physical activity questionnaire	**Liver cancer incidence**		Age
					Low	1	
					Moderate to high	0.85 (0.79, 0.92)	
